# Differential Inflammatory Cytokine Elaboration in Serum from Brick Kiln Workers in Bhaktapur, Nepal

**DOI:** 10.3390/diseases12060129

**Published:** 2024-06-17

**Authors:** Katrina L. Curtis, Ashley Chang, James D. Johnston, John D. Beard, Scott C. Collingwood, James D. LeCheminant, Neil E. Peterson, Andrew J. South, Clifton B. Farnsworth, Seshananda Sanjel, Benjamin T. Bikman, Juan A. Arroyo, Paul R. Reynolds

**Affiliations:** 1 Department of Cell Biology and Physiology, Brigham Young University, Provo, UT 84602, USA; 2 Department of Public Health, Brigham Young University, Provo, UT 84602, USA; 3 Department of Pediatrics, University of Utah, Salt Lake City, UT 84112, USA; 4 Rocky Mountain Center for Occupational and Environmental Health, University of Utah, Salt Lake City, UT 84112, USA; 5 Department of Nutrition, Dietetics, and Food Science, Brigham Young University, Provo, UT 84602, USA; 6 College of Nursing, Brigham Young University, Provo, UT 84602, USA; 7 Department of Civil and Construction Engineering, Brigham Young University, Provo, UT 84602, USA; 8 Department of Community Medicine and Public Health, Karnali Academy of Health Sciences, Jumla 21200, Nepal

**Keywords:** cytokines, particulates, lung, chronic inflammation, systemic inflammation

## Abstract

Previous studies involving workers at brick kilns in the Kathmandu Valley of Nepal have investigated chronic exposure to hazardous levels of fine particulate matter (PM_2.5_) common in ambient and occupational environments. Such exposures are known to cause and/or exacerbate chronic respiratory diseases, including chronic obstructive pulmonary disease (COPD) and asthma. However, there is a paucity of data regarding the status of systemic inflammation observed in exposed workers at brick manufacturing facilities within the country. In the current study, we sought to elucidate systemic inflammatory responses by quantifying the molecular cytokine/chemokine profiles in serum from the study participants. A sample of participants were screened from a kiln in Bhaktapur, Nepal (*n* = 32; 53% female; mean ± standard deviation: 28.42 ± 11.47 years old) and grouped according to job category. Blood was procured from participants on-site, allowed to clot at room temperature, and centrifuged to obtain total serum. A human cytokine antibody array was used to screen the inflammatory mediators in serum samples from each of the participants. For the current study, four job categories were evaluated with *n* = 8 for each. Comparisons were generated between a control group of administration workers vs. fire master workers, administration workers vs. green brick hand molders, and administration workers vs. top loaders. We discovered significantly increased concentrations of eotaxin-1, eotaxin-2, GCSF, GM-CSF, IFN-γ, IL-1α, IL-1β, IL-6, IL-8, TGF-β1, TNF-α, and TIMP-2 in serum samples from fire master workers vs. administration workers (*p* < 0.05). Each of these molecules was also significantly elevated in serum from green brick hand molders compared to administration workers (*p* < 0.05). Further, each molecule in the inflammatory screening with the exception of TIMP-2 was significantly elevated in serum from top loaders compared to administration workers (*p* < 0.05). With few exceptions, the fire master workers expressed significantly more systemic inflammatory molecular abundance when compared to all other job categories. These results reveal an association between pulmonary exposure to PM_2.5_ and systemic inflammatory responses likely mediated by cytokine/chemokine elaboration. The additional characterization of a broader array of inflammatory molecules may provide valuable insight into the susceptibility to lung diseases among this population.

## 1. Introduction

The manufacturing of bricks is a major industry in the Kathmandu Valley of Nepal, primarily due to the fact that bricks represent the main building material employed throughout the country [1]. The number of brick kilns in the Kathmandu Valley is estimated to be about 120 [2], and the impact of personal air pollution generated at brick kilns on the pulmonary status of its diverse workforce has been the subject of investigation for many years [3]. While the region is responsible for the production of nearly 600 million bricks per year, the relatively small size of the region, the valley’s topography, and general proximity to neighboring kilns makes the area of key interest in the characterization of exposure to particulates [2]. Kiln workers are particularly at risk due to the prevalence of respirable particulate matter with aerodynamic diameters of 4.0 μm or smaller, such as crystalline silica and iron oxide, prevalent in the clay commonly used during the manufacturing process [4]. Combined with these and other occupational hazards, workers are additionally exposed to PM_2.5_ abundantly available from the brick-making process, abundant fugitive dust, vehicular exhaust, regional transport of pollutants, and the regular burning of biomass [5]. It is clear that the Nepali brick worker experiences abundant exposure to personal and general environmental air pollution during the course of their employment.

The inhalation of PM_2.5_ underpins the most significant environmental health issues worldwide [6]. Exposure has been implicated in the induction and/or exacerbation of myriad pathologies including carcinogenesis [7], cardiovascular diseases [8], metabolic syndromes [9], pregnancy complications [10], and of course, a host of lung diseases [11]. Due primarily to the intimate apposition of the pulmonary apparatus with the environment, the respiratory system is particularly compromised by exposure to PM_2.5_, which leads to symptoms including persistent cough, shortness of breath, wheezing, and chronic phlegm production. These symptoms are common during the progression of chronic obstructive pulmonary disease (COPD). COPD is a respiratory disorder that is considered an irreversible condition in which chronic bronchitis and emphysema target the tissues comprising the lung parenchyma. Unfortunately for sufferers, treatment is limited to strategies that relieve symptoms and diminish acute exacerbations of the disease. Disease progression occurs primarily due to long-term exposure to particulates generated during the combustion of various tobacco products, but exacerbation is also associated with long-term exposure to ambient air pollution and fine biomass particulates [12]. Such observations reveal that while the major risk factor for developing COPD remains exposure to primary tobacco smoking, approximately one-quarter of all diagnosed patients with COPD are never smokers [13,14,15,16].

During the initiation and progression of COPD, individuals experience the molecular effects of marked inflammatory signaling, usually under the control of excessive activation of NF-κB. NF-κB is a key regulator of inflammatory molecule secretion and its upregulation coincides with pathological responses to cell perturbation following exposure to myriad stimuli. Such responses include, but are not limited to, prolonged lung inflammation, dysregulated protease production, and mucus hypersecretion that lead to impaired lung function coincident with alveolar destruction, decreased gas exchange, and obstructed airflow [17]. The abundant pulmonary secretion of inflammatory molecules invariably penetrates the systemic circulatory system and manages organismal responses to stimuli. As such, an extended consequence of pulmonary exposure to PM_2.5_ is the activation of systemic inflammation. Abundant studies demonstrate the connection between ambient air pollution exposure and adverse systemic health effects orchestrated by oxidative stress [18], chronic inflammation [19], and abnormal immune regulation [20]. Notable systemic inflammation stemming from PM_2.5_ exposure can be reflected by serum cytokine levels and altered leukocyte counts [21]. 

Augmentation of pro-inflammatory cytokines and the cells that secrete them are implicated in the regulation of the biological inflammatory and immune responses observed in numerous disease states. For instance, particulate air pollution is known to increase the levels of various inflammatory molecules which are implicated in both acute and chronic health effects. Exposure to fine particulate matter (PM_2.5_ and PM_10_) has been consistently associated with increased production of pro-inflammatory cytokines such as tumor necrosis factor-alpha (TNF-α), interleukins (IL-1β, IL-6, IL-8), and granulocyte–macrophage colony-stimulating factor (GM-CSF), contributing to systemic inflammation and increased risk of cardiovascular and respiratory diseases [22]. In addition, particulate matter can induce oxidative stress in respiratory tract cells, leading to further inflammation and injury. This oxidative stress is marked by increased levels of reactive oxygen species (ROS) and is strongly associated with the activation of signaling pathways such as NF-κB, which further drives the production of inflammatory cytokines [23]. Moreover, ambient PM exposure is linked to endothelial dysfunction, reflected by elevated levels of endothelial microparticles and inflammatory markers like soluble intercellular adhesion molecule 1 (sICAM-1) and vascular cell adhesion molecule 1 (sVCAM-1), indicating a systemic inflammatory response. These findings collectively demonstrate that particulate air pollution induces a broad spectrum of inflammatory responses across different organ systems, potentially explaining the diverse array of health issues associated with poor air quality. Therefore, the current investigation was designed to quantify the elaboration of inflammatory mediators in the serum of Nepali brick kiln workers in order to foreshadow subsequent research into the molecular mechanisms of inflammation. A clearer understanding of the mechanisms that perpetuate and worsen disease should be beneficial in identifying possible targets that may mitigate the inflammatory excursions common in this population. Additionally, while the scope of this specific study was to assess the systemic inflammatory profiles of the study participants, a natural subsequent endeavor that correlates NF-κB activity in exposed lung tissue and systemic inflammation would be highly informative.

## 2. Methods and Materials

### 2.1. Study Design

**Figure 1:**
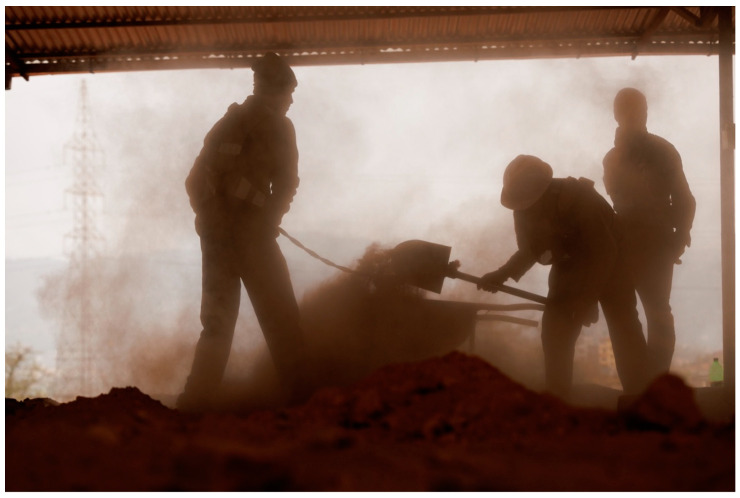
Brick kiln workers in Bhaktapur, Nepal categorized as top loaders, March 2023. Photograph by Jaren Wilkey, University Communications, Brigham Young University, Provo, UT USA.

This study was designed as a cross-sectional, observational study that aimed to characterize health status, exposure burden to respirable crystalline silica and PM_2.5_ [24], and to obtain serum from brick workers in Bhaktapur, Nepal. The manufacture of bricks in the Kathmandu Valley occurs during the dry season—from November to March. Samples from participants (*n* = 32, Table 1) were procured from 13 to 20 March 2023 at a coal-fed kiln involving a fan that pulls hot air and flue gasses across bricks stacked in a manner that moves the air in a zigzag pattern. This brick patterning increases heat distribution and air contact time, which results in higher brick quality and fuel efficiency [25,26]. Participants were selected based on convenience sampling and written informed consent was obtained from all participants prior to commencing the study by using native-language translations and on-site bi-lingual interpreters to answer any questions. All participants were adult employees of the brick kiln primarily sampled from four job categories: administration (management and office employees), fire masters (workers who added coal to bricks during the firing process), green brick hand molders (workers that formed green bricks using hand molds), and top loaders (Figure 1; workers who removed soil from the top of the brick kiln after firing had been completed). Workers in each category experienced temporal exposure trends to PM_2.5_ coincident with diverse dust/silica species [24]. For this particular study, a sample size of 8 for each of the 4 job categories was evaluated, with equal representation by age for each job description (Table 1). The study was reviewed and approved by the Institutional Review Boards (IRBs) at Brigham Young University (BYU) and the Nepal Health Research Council (NHRC).

### 2.2. Blood Sampling

Whole blood was collected in a vacutainer SST tube (Cat# 367986; BD Biosciences, Lakes, NJ, USA) and immediately inverted 4–5 times. Blood samples were then allowed to coagulate upright for at least 30 min and stored at 2–8 °C. The samples were centrifuged at 1200 RCF for 10 min before the upper serum supernatant was removed into clean 15 mL serological tubes. Aliquots of 0.5 mL were then placed into fresh polypropylene microfuge tubes and shipped on ice prior to quantifying inflammatory molecule abundance as described in the analyses below. 

### 2.3. Inflammatory Molecule Analyses

The protein concentration of each serum sample was quantified using a bicinchoninic acid assay (BCA) Protein Assay Kit (Thermo Fisher Scientific, Waltham, MA, USA). A serum sample that contained 125 μg of protein was collected from each study participant (a total of *n* = 8 per job category), and divided to create two pools, each with a concentration of 500 μg/mL (serum samples from 4 participants in the same job category were pooled for each blot; *n* = 4 per blot). These two sample pools per job category were added to individual membranes from a human inflammation antibody array (Abcam, Waltham, MA, USA) and allowed to incubate overnight. Biotinylated antibodies were then added to each membrane and incubated overnight followed by a final incubation with a streptavidin-conjugated fluorescent antibody (Cat# S32358, Thermo Fisher Scientific) to detect cytokine protein expression. Membranes were imaged using the Odyssey DLx Near-Infrared Fluorescence Imaging System (LI-COR, Lincoln, NE, USA). Blot intensities were quantitatively analyzed using the publicly available Image J software (Version 1.54, U.S. National Institutes of Health, Bethesda, MA, USA) [27] and normalized to the blot’s positive controls included on each membrane as outlined in the manufacturer’s instructions. Fold inductions were then calculated by comparing the ratios observed in the administration group to the ratios detected from the other job categories. 

### 2.4. Statistical Analysis

Mean values ± standard deviation per group were assessed by one- and two-way analyses of variance (ANOVA) and those with *p* values < 0.05 were considered significant. Statistical analyses were performed with GraphPad Prism, version 7.0.

## 3. Results

### 3.1. Expression Profiles for Eotaxin-1, Eotaxin-2, GCSF, and GM-CSF

**Figure 2:**
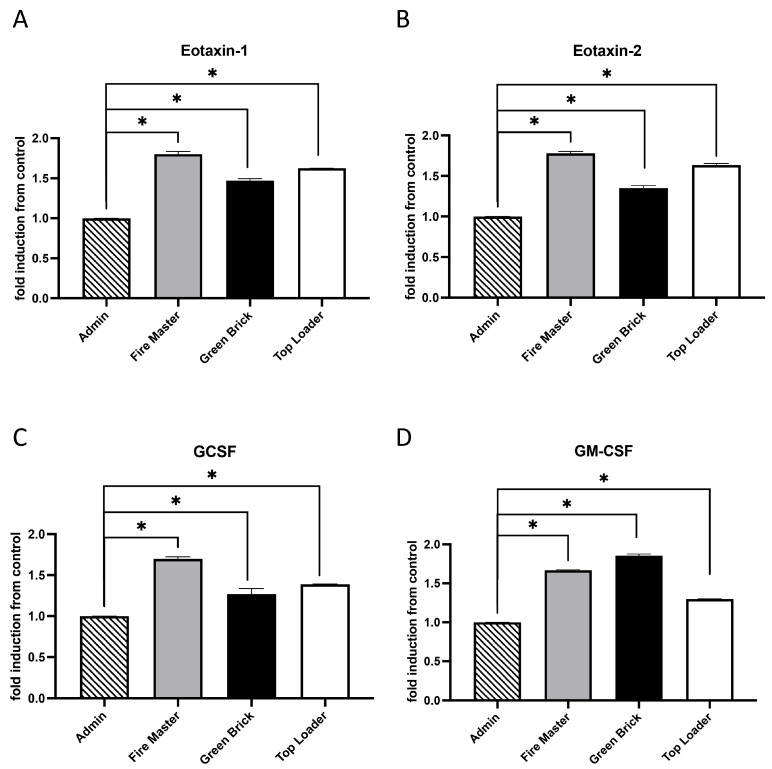
Cytokine/chemokine expression in serum samples from brick kiln workers in Bhaktapur, Nepal, March 2023. Inflammatory mediators were screened in pooled serum samples obtained from study participants from each job category. Compared to administrative personnel, there was significantly more Eotaxin-1, Eotaxin-2, GCSF, and GM-CSF detected in serum from fire masters, green brick hand molders, and top loaders (**A**–**D**). Significant differences in mediator abundance are noted as * *p* ≤ 0.05.

Eotaxin-1 (CCL11) and Eotaxin-2 (CCL24) are chemokines that act through the CCR3 receptor primarily involved in the recruitment of eosinophils to sites of inflammation [28]. Granulocyte colony-stimulating factor (G-CSF) is crucial for the proliferation, differentiation, and survival of neutrophil precursors and for enhancing the effector functions of mature neutrophils [29]. Granulocyte–macrophage colony-stimulating factor (GM-CSF) is a cytokine that affects the production and function of both granulocytes and macrophages. It has a broader range of action compared to G-CSF, influences the differentiation of myeloid progenitor cells into various types of immune cells, and enhances the functional abilities of mature immune cells [30]. We discovered that Eotaxin-1, Eotaxin-2, GCSF, and GM-CSF were each upregulated in fire master workers (1.51-fold, 1.54-fold, 1.42-fold; 1.51-fold; all *p* < 0.05), green brick hand molders (1.31-fold, 1.27-fold, 1.20-fold, 1,2-fold; all *p* < 0.05), and top loaders (1.41-fold, 1.60-fold, 1.30-fold, 1.30-fold; all *p* < 0.05) when compared to the group of administration workers (Figure 2A–D).

### 3.2. Expression Profiles for IFN-γ, TNF-α, IL-1α, and IL-1β

**Figure 3:**
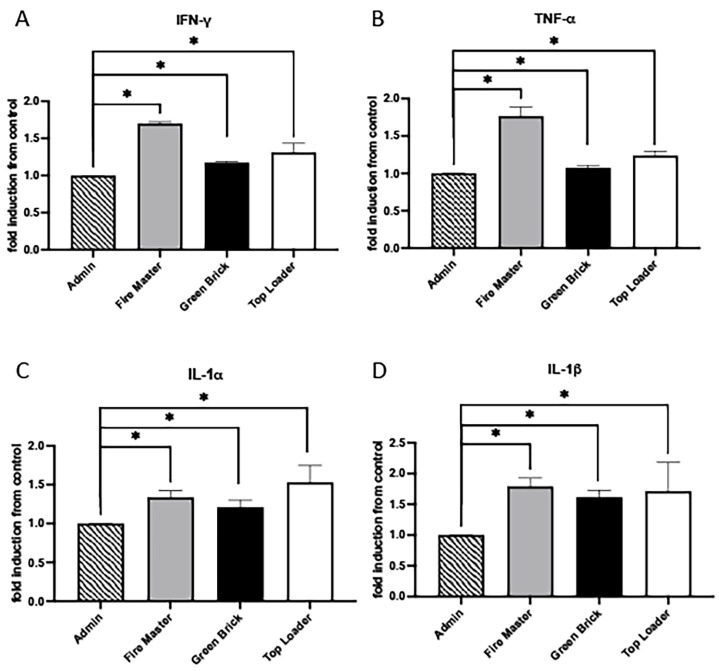
Cytokine/chemokine expression in serum samples from brick kiln workers in Bhaktapur, Nepal, March 2023. Inflammatory mediators were screened in pooled serum samples obtained from study participants from each job category. Compared to administrative personnel, there was significantly more IFN-g, TNF-a, IL-1a, and IL-1b detected in serum from fire masters, green brick hand molders, and top loaders (**A**–**D**). Significant differences in mediator abundance are noted as * *p* ≤ 0.05.

Interferon-gamma (IFN-γ), Tumor Necrosis Factor-alpha (TNF-α), Interleukin-1 alpha (IL-1α), and Interleukin-1 beta (IL-1β) are key cytokines in the immune system that play pivotal roles during inflammatory responses. IFN-γ is primarily produced by T cells and natural killer (NK) cells and is essential for innate and adaptive immunity against infections. It activates macrophages, enhances the presentation of antigens, and promotes the differentiation of T cells into Th1 cells [31]. TNF-α is produced by macrophages, T cells, and NK cells. It has a wide range of pro-inflammatory actions and is involved in the regulation of immune cells, induction of fever, apoptotic cell death, and inflammation [32]. IL-1α and IL-1β are closely related cytokines produced mainly by activated macrophages and both have been implicated in a variety of cellular activities, including cell proliferation, differentiation, and apoptosis [33]. The relationship among IFN-γ, TNF-α, IL-1α, and IL-1β is characterized by their synergistic interactions in immune responses. We discovered that IFN-γ, TNF-α, IL-1α, and IL-1β were each upregulated in fire master workers (1.71-fold, 1.70-fold, 1.34-fold, 1.80-fold; all *p* < 0.05), green brick hand molders (1.2-fold, 1.10-fold, 1.212-fold, 1.62-fold; all *p* < 0.05), and top loaders (1.31-fold, 1.24-fold, 1.53-fold, 1.71-fold; all *p* < 0.05) when compared to the group of administration workers (Figure 3A–D).

### 3.3. Expression Profiles for IL-6, IL-8, TGF-b1, and TIMP-2

**Figure 4:**
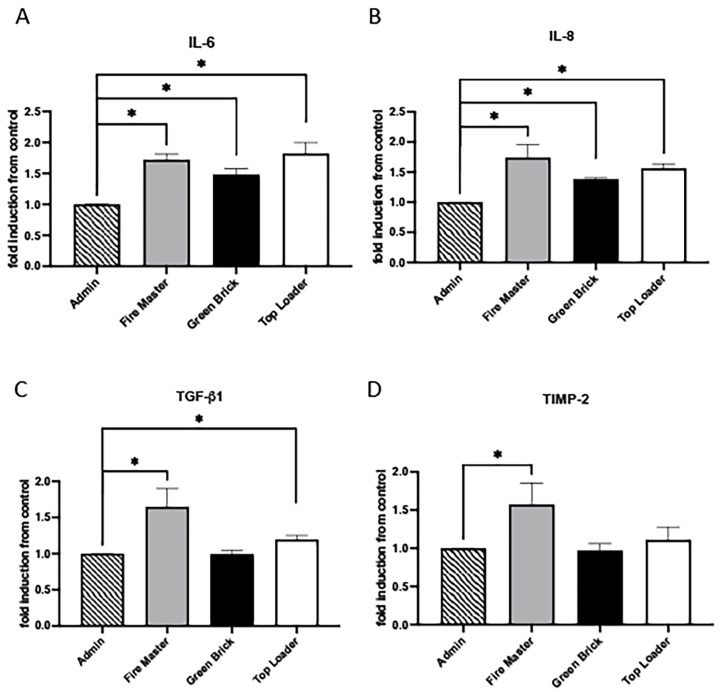
Cytokine/chemokine expression in serum samples from brick kiln workers in Bhaktapur, Nepal, March 2023. Inflammatory mediators were screened in pooled serum samples obtained from study participants from each job category. Compared to administrative personnel, there was significantly more IL-6 and IL-8 detected in serum from fire masters, green brick hand molders, and top loaders (**A**,**B**). TGF-b1 was significantly increased in serum from fire master workers and top loaders compared to administration workers but unchanged in green brick hand molders (**C**). TIMP-2 was significantly increased in fire master workers compared to administrative personnel but unchanged in green brick hand molders or top loaders (**D**). Significant differences in mediator abundance are noted as * *p* ≤ 0.05. TGF-b1.

IL-6 and IL-8 are involved in the initiation and propagation of inflammatory responses, with IL-6 acting more broadly in inducing fever and acute-phase responses, while IL-8 specifically recruits neutrophils to sites of infection or injury [34,35]. TGF-β1 and TIMP-2 have countering effects on matrices and are crucial in the tissue repair and remodeling processes observed during inflammatory responses [36,37]. IL-6 and IL-8 were each upregulated in fire master workers (1.72-fold, 1.74-fold; each *p* < 0.05), green brick hand molders (1.50-fold, 1.40-fold; each *p* < 0.05), and top loaders (1.82-fold, 1.60-fold; each *p* < 0.05; Figure 4A,B). Compared to administration worker controls, TGF-β1 was significantly increased in fire master workers (1.65-fold and *p* < 0.05) and top loaders (1.20-fold and *p* < 0.05) but unchanged in the green brick hand molders (Figure 4C). TIMP-2 was significantly elevated in fire master workers vs. administration workers (1.60-fold and *p* < 0.05) and unchanged when comparing green brick hand molders or top loaders to administration workers (Figure 4D).

## 4. Discussion

The pulmonary system is positioned as an expansive yet delicate interface between the organism and its surrounding environment. As such, exposure to deleterious stimuli such as silica, fine brick dust, or other PM_2.5_ can elicit cellular responses following interactions with lung parenchyma. In this study, we investigated the association between job categories at the brick kiln that experienced high levels of particulate exposures and changes in serum levels of a broad panel of cytokines and chemokines, including eotaxin-1, eotaxin-2, G-CSF, GM-CSF, IFN-γ, IL-1α, IL-1β, IL-6, IL-8, TGF-β1, TNF-α, and TIMP-2. Our findings suggest that working in a job that involves exposure to PM_2.5_ is significantly correlated with alterations in the serum concentrations of these immunological mediators that function during inflammatory responses. Accordingly, inflammatory mediator expression in serum would further indicate a systemic inflammatory response and the general modulation of immune functions in exposed individuals.

The increase in pro-inflammatory cytokines such as IL-6, TNF-α, and IL-1β following PM_2.5_ exposure is consistent with previous research that indicated that air pollution can trigger systemic inflammation [38,39]. IL-6, a key mediator of fever and acute-phase responses, has been shown to rise in conditions of environmental stress and contribute to the pathogenesis of cardiovascular and pulmonary diseases [40]. Similarly, TNF-α and IL-1β are critical in mediating inflammatory and immune responses, and their elevated levels may reflect an enhanced state of immune activation in response to particulate matter inhalation [41]. These three cytokines are central to the inflammatory mechanisms that contribute to both the pulmonary and systemic effects of air pollution. Studies have demonstrated that exposure to particulate matter activates alveolar macrophages which then release TNF-α and IL-1β. These cytokines not only stimulate other cells to produce additional cytokines including IL-6 but also enhance the expression of various inflammatory mediators, amplifying the inflammatory response in the lung tissue [42]. Additionally, IL-1β and TNF-α have been shown to influence the production of IL-6 in cardiac cells, suggesting systemic effects beyond the lungs, particularly impacting cardiovascular health [43]. Moreover, TNF-α and IL-1β have been found to increase the permeability of endothelial and epithelial barriers, further contributing to inflammation and potential tissue damage [44]. Accordingly, TNF-α, IL-1β, and IL-6 play critical roles in mediating the inflammatory response to particulate air pollution, with effects that may extend from the pulmonary system to impact systemic health.

The observed increase in the chemokines eotaxin-1 and eotaxin-2 further supports the hypothesis that PM_2.5_ exposure leads to the chemotactic recruitment of eosinophils and contributes to allergic inflammation and potentially exacerbates conditions such as asthma [45]. This is particularly relevant in environments common in the Kathmandu Valley, including at brick kilns, where air pollution levels are high, and respiratory conditions are likely more prevalent [46]. Furthermore, our results indicated elevated levels of G-CSF and GM-CSF following PM_2.5_ exposure, which are crucial for the proliferation and differentiation of granulocyte and monocyte lineages [29,30]. This discovery suggests a plausible compensatory mechanism to replenish immune cells consumed in the response to inhaled particulates.

The analysis of IFN-γ, a key cytokine in antiviral immunity, showed an increase that could signify an enhanced state of immune readiness against infections that is possibly due to the immune system’s recognition of PM_2.5_ as a pathogenic stimulus [31]. However, the upregulation of TGF-β1 and TIMP-2, involved in tissue repair and fibrosis, raises concerns about chronic exposure leading to adverse tissue remodeling and fibrotic diseases [36,37]. 

We also observed an increase in IL-8 levels in serum samples following exposure to PM_2.5_, which suggests a direct link between particulate matter pollution and the activation of inflammatory pathways that involve the recruitment of neutrophils to sites of inflammation. Studies, such as that by Yan et al. [47], have demonstrated that exposure to PM_2.5_ can lead to an upregulation of IL-8 expression in both respiratory epithelial cells and macrophages, which indicates a mechanism through which air pollution exacerbates pulmonary inflammation and potentially contributes to the development of respiratory conditions such as asthma and COPD [48]. Additional studies indicate that exposure to particulate matter and diesel exhaust particles (DEPs) can significantly upregulate IL-8 expression in human airway epithelial cells. This induction of IL-8 is mediated through mechanisms involving the activation of NF-κB and is influenced by the presence of transition metals like iron within the PM, which can catalyze the production of reactive oxygen species (ROS), further enhancing IL-8 expression [49]. The elevation of IL-8 following PM_2.5_ exposure highlighted the potential role of IL-8 in immune responses that attempt to counteract the harmful effects of inhaled particulates.

This study underscores the intricate relationship between air pollution and systemic immune responses. The observed alterations in cytokine and chemokine levels following PM_2.5_ exposure highlight the potential of particulate matter to influence a wide array of immune processes, from inflammation to cell recruitment and tissue remodeling. While the results clearly demonstrate differential systemic inflammatory responses among each of the employment categories at the brick kiln, important additional work remains. For example, subsequent studies should further distinguish the extent of Th1 and Th2 responses. Th1 and Th2 cells represent two distinct subsets of CD4+ T helper cells that play critical roles in the immune system’s response to different pathogens and in immunological homeostasis. Th1 cells are primarily involved in promoting cell-mediated immunity and are key players in the defense against intracellular pathogens such as viruses and certain bacteria. They produce a characteristic set of cytokines we observed in serum from kiln workers such as IFN-g. Additional Th1-related molecules include IL-2 and TNF-b, which are crucial for activating macrophages and cytotoxic T cells that kill infected host cells or pathogens. On the other hand, Th2 cells facilitate humoral immunity and are pivotal in the immune response to extracellular entities. Th2 cells produce a different set of cytokines, including IL-4, IL-5, IL-6, IL-9, IL-10, and IL-13, which stimulate B-cell differentiation into plasma cells and IgE production, and recruit eosinophils to inflammatory sites. These cytokines also help suppress the macrophage-activating effects of Th1 cytokines, thus skewing the immune response toward a humoral response rather than a cell-mediated response. A follow-up evaluation aimed at discerning the balance between Th1 and Th2 responses is crucial for understanding immune homeostasis. Dysregulation in Th1/Th2 balance can lead to various pathological conditions, where a dominance of Th1 responses is often associated with chronic inflammatory and autoimmune diseases, and a dominance of Th2 responses can lead to allergic diseases. Careful consideration of these points may further clarify environmental factors and genetic predispositions that influence the differentiation and function of T helper subsets, impacting the overall immune response in an individual.

The findings of this current investigation provide an important initial step in understanding the plausible mechanisms underlying the health impacts of particulate exposure. They also contribute vital endpoints that foreshadow inflammatory signaling anomalies and pathways that exacerbate the effects of exposure among key job categories at brick kilns. These and other similar research pursuits underscore the importance of reducing exposure to mitigate adverse human health effects. However, this study has limitations due to the sample size of the participants screened at the kiln. The sample size of *n* = 8 per group may not fully capture the variability in the serum concentrations of inflammatory cytokines, potentially leading to less-generalizable results. Despite significant differences observed for the 12 screened cytokines when comparing the job category groups, increasing the sample size would enhance statistical power and further confirm applicability.

## 5. Conclusions

The working conditions of brick kiln workers in the Kathmandu Valley of Nepal are exacerbated by exposure to fine particulate matter. The current study involved the evaluation of a broad panel of inflammatory cytokines in the serum of kiln workers. The panel revealed marked upregulation of systemic inflammation mediated by pro-inflammatory cytokine/chemokine elaboration and the job category of fire master representing the highest inflammatory molecule abundance. While this study was limited by sample size, a follow-up visit to the brick kiln is planned and the subsequent study will include a much larger sample size necessary for validating the results. These discoveries reveal a probable link between pulmonary exposure to particulates and systemic inflammatory responses.

## Figures and Tables

**Table 1:** Demographic information for workers at a brick kiln in Bhaktapur, Nepal, March 2023.

Characteristic	*n*	%
Total	32	100
Age at interview (years)		
19–22	4	13
>22–28	8	25
>28–34	8	25
>34–40	8	25
>40–61	4	13
Median (IQR)	28.42 (11.37)	
Sex or gender		
Male	15	48
Female	17	52

Interquartile range (IQR).

## Data Availability

Data and other materials are available from the corresponding author on reasonable request.
